# Relation of time in range to severity of coronary artery disease in patients with type 2 diabetes: A cross-sectional study

**DOI:** 10.1515/med-2025-1158

**Published:** 2025-06-04

**Authors:** Weiguo Ma, Jinfeng Li, Xiaojuan Shao, Xiaohong Yin, Chuanqing Xie, Fenfen Wang, Ya Li

**Affiliations:** Department of Endocrinology, The First Affiliated Hospital of Xi’an Medical University, Xi’an, 710077, China; Department of Endocrinology, The First Affiliated Hospital of Xi’an Medical University, 48 Fenghao West Road, Lianhu District, Xi’an, 710077, China

**Keywords:** continuous glucose monitoring, coronary artery disease, time in range, type 2 diabetes, surrogate marker

## Abstract

**Background:**

Despite the high prevalence and serious clinical implications of coronary artery disease (CAD) in patients with type 2 diabetes mellitus (T2DM), the relationship between glycemic control and CAD is usually overlooked. This study aimed to explore the relationship between time in range (TIR), a surrogate marker for glycemic control, and CAD in patients with T2DM.

**Methods:**

Overall, 334 patients with T2DM were included and analyzed in this cross-sectional study. The presence of CAD was determined angiographically and the Gensini score was applied to evaluate CAD severity. TIR was calculated from sensor glucose from continuous glucose monitoring. Multivariable-adjusted logistic regression analysis was used to evaluate the relationship between TIR and CAD presence.

**Results:**

T2DM with CAD had significantly lower TIR than those without (75.68 ± 13.74 vs 66.12 ± 11.87, *P* < 0.01). Moreover, TIR was correlated with CAD severity as indicated by the Gensini score. Multivariable-adjusted logistic regression analysis indicated that a higher TIR was an independent protective factor for CAD in patients with T2DM (OR = 0.919, 95% CI: 0.896–0.942).

**Conclusion:**

TIR is significantly and independently related to CAD severity in T2DM patients. Thus, TIR could be a promising biomarker for the noninvasive assessment of CAD presence and severity in T2DM.

## 1 Introduction

The absolute number of patients with diabetes mellitus worldwide has quadrupled in the past three decades, rendering it as the ninth major cause of mortality. It is estimated that type 2 diabetes mellitus (T2DM) accounts for approximately 90% of all diabetes mellitus cases that occurs in 1 in 11 adults [1]. By 2040, the global prevalence of diabetes is projected to exceed 640 million [2]. Alarmingly, diabetic patients have a 2–3-fold increased risk of major adverse cardiovascular events, with over two-thirds of these patients succumbing to these complications [3]. This rising cardiovascular burden has placed a significant strain on healthcare systems [4,5]. Hemoglobin A1c (HbA1c) has traditionally been used as a surrogate marker to assess glycemic control [6]. A previous study has demonstrated that maintaining HbA1c levels below 7% is associated with a reduced risk of myocardial infarction [7]. There is a strong correlation between HbA1c levels and cardiovascular mortality; a 1% increase in HbA1c is associated with a 1.15-fold higher risk of cardiovascular death [8]. Currently, the close relationship between elevated HbA1c and cardiovascular complications, as well as all-cause mortality among T2DM patients, has been firmly established [9].

While glycemic control has been suggested to be essential for the reduction of mortality in patients with T2DM, the relationship between HbA1c and glycemic control is increasingly being questioned. Some studies have observed discordance between HbA1c and parameters like fasting blood glucose (FPG), mean blood glucose or continuous glucose monitoring (CGM) [10,11]. In comparison, with the advancement of CGM technology, the metric of time in range (TIR) is increasingly regarded as a new standard for glycemic control. TIR is defined as the time or percentage of glucose in the target range (usually 3.9–10.0 or 3.9–7.8 mmol/L) within 24 h [12]. Studies have revealed that TIR could serve as a surrogate biomarker for the urgency of hypo- and hyper-glycemic events and be individually personalized to meet clinical demands [13,14]. For instance, a TIR of 70% (70–180 mg/dL [3.9–10.0 mmol/L]) corresponds to an HbA1c of approximately 7.0%, while a TIR of 50% corresponds to an HbA1c of about 8.0% [15]. Furthermore, each 10% increase in TIR is associated with an approximate 0.5% improvement in HbA1c levels [16]. A cross-sectional study has found negative associations between TIR and the prevalence of diabetic retinopathy and carotid intima-media thickness [17]. Beck et al. also identified significant correlations between TIR and the development of diabetic retinopathy and microalbuminuria [18]. Additionally, a large-scale cohort study has suggested that the TyG index could assist in risk stratification for glycemic management in diabetic patients with coronary artery disease (CAD) [19]. Although prior evidence indicates a possible link between TIR and diabetic complications [17–19], the exact association between TIR and CAD in T2DM patients remains largely unknown. Thus, the purpose of the present study is to explore such correlations to further provide a basis for the early diagnosis and evaluation of cardiovascular events.

## 2 Materials and methods

### 2.1 Study subjects

Patients with T2DM undergoing coronary angiography between January 2018 and July 2020 at the Department of Endocrinology, The First Affiliated Hospital of Xi’an Medical University were included. Inclusion criteria were adult patients (age ≥ 18 years) with T2DM on a stable glucose-lowering regimen over the past 3 months. Diabetes mellitus was diagnosed in accordance with the 1998 WHO diagnostic criteria [20]. Exclusion criteria were presence of severe complications, such as hyperglycemic hyperosmolar state, diabetic ketoacidosis, or hypoglycemic events within the past 3 months; non-T2DM, such as T1DM; presence of malignancy or severe hepato-renal dysfunction; use of steroid within the previous 3 months; and concomitant thyroid dysfunction, infections, or previous coronary artery interventions. Overall, 334 patients with T2DM undergoing coronary angiography were included, including 64 patients without CAD (control group) and 270 patients with CAD (CAD group).

### 2.2 Clinical data and laboratory measurement

Hypertension was considered for those with a blood pressure ≥140/90 mmHg or currently taking any antihypertensive medications. Body mass index (BMI) was measured as weight (kg) divided by height squared (m^2^). Smoking history and information on current use of medications were collected by interviews during the medical examinations.

Blood samples and fresh first-void morning urine samples were collected after a 12 h overnight fast and analyzed for fasting blood glucose (FPG), total cholesterol (TC), triglycerides (TG), low-density lipoprotein cholesterol (LDL-C), high-density lipoprotein cholesterol (HDL-C), apolipoprotein A (Apo A), apolipoprotein B (Apo B), apolipoprotein E (Apo E), creatinine (Cr), blood urea nitrogen (BUN), uric acid (UA), urinary albumin/creatinine concentration, fasting insulin (FINS), and HbA1C using an automated biochemical analyzer (Hitachi 7600–110; Hitachi, Tokyo, Japan). Besides, the visit-to-visit variability of FPG was evaluated using two variability measures, calculating the following for individual participants: (1) the standard deviation (SD) and (2) the coefficient of variation (CV), calculated by SD divided by mean.

### 2.3 TIR measurement

All participants underwent CGM (Abbott Freestyle Libre Pro; FGM, USA) for 72 h. During the CGM period, participants adhered to a standardized diet and their initial hypoglycemic regimen. Specifically, the CGM sensor was inserted on the first day of admission (Day 0) and removed after 72 h, providing a daily record of 288 continuous sensor readings. TIR was defined as the percentage of time within a 24 h period that glucose levels were within the target range of 3.9–10.0 mmol/L. Following the 3-day monitoring period, various metrics were calculated using previously reported methods [21], including TIR, time above range (TAR), time below range (TBR), the mean amplitude of glycemic excursions (MAGE), SD, the mean of daily differences (MODD), and the glucose CV.

### 2.4 Coronary angiography

Severe CAD was considered in the presence of ≥50% stenosis of at least one coronary artery. The method for Gensini score is the same as previously reported [22]. Patients were divided into three groups based on the tertile of the Gensini score: Group 1, with Gensini score ≤11; Group 2, with Gensini score in the range of 12–36; Group 3, with Gensini score >36.

### 2.5 Statistical analysis

Statistical analysis was conducted using SPSS software. Continuous variables with normal distribution are presented as mean ± SD, while those with non-normal distribution are expressed as median (interquartile range, 25–75%). Categorical variables are presented as percentages. For continuous variables, intergroup comparisons were performed using Student’s *t*-test if they followed a normal distribution; otherwise, the Mann–Whitney *U*-test was used. For categorical variables, intergroup comparisons were conducted using the chi-square test (*χ*² test). A *P* value <0.05 was considered statistically significant.


**Informed consent:** Written informed consent was obtained from all participants.
**Ethical approval:** The study was approved by the Ethics Committees of The First Affiliated Hospital of Xi'an Medical University and conducted in accordance with the principles of the Declaration of Helsinki.

## 3 Results

As compared to the control group, patients in the CAD group are older, have a higher BMI, longer duration of T2DM and higher systolic blood pressure (SBP), a higher prevalence of current smoking, and higher insulin use (Table 1). In terms of laboratory data, CAD group was associated with significantly elevated TC, LDL-C, ApoB, ApoE, urinary albumin/creatinine concentration, BUN, serum creatinine, UA, HbA1c, TAR% of >10.0 mmol/L, lower TIR, and lipoprotein (Lpa) (*P* < 0.05). No significant differences were observed between the two groups in terms of gender, diastolic blood pressure (DBP), TG, HDL-C, ApoA, FINS, SD, MODD, MAGE, TBR% of <3.9 mmol/L, and use of oral hypoglycemic agents.

**Table 1:** Comparison of subjects in non-CAD and CAD groups

Variables	Non-CAD (*n* = 64)	CAD (*n* = 270)	*P* value for trend
Gender (%men)	51.25	52.61	0.312
Age (years)	54.63 ± 10.12	59.56 ± 11.04	<0.001
Diabetes duration (years)	5.14 ± 2.74	10.51 ± 5.21	<0.001
SBP (mmHg)	124.24 ± 24.69	130.10 ± 15.27	0.032
DBP (mmHg)	75.05 ± 17.09	76.54 ± 8.26	0.301
BMI (kg/m^2^)	23.71 ± 2.89	25.18 ± 3.64	0.020
Current smoker (%)	23.15	32.34	<0.001
Total cholesterol (mmol/L)	4.07 ± 1.38	4.51 ± 1.60	0.029
TG (mmol/L)	1.43 (1.01,1.34)	1.51 (1.03,2.15)	0.697
LDL-C (mmol/L)	2.47 ± 0.96	2.85 ± 0.86	0.017
HDL-C (mmol/L)	1.19 ± 0.32	1.04 ± 0.52	0.273
ApoA (mmol/L)	1.14 ± 0.21	1.15 ± 0.24	0.752
ApoB (mmol/L)	0.90 ± 0.32	1.01 ± 0.29	0.025
ApoE (mmol/L)	39.21 ± 18.01	43.97 ± 17.72	<0.001
LPa (mmol/L)	124 (47.13, 281)	106 (48.67, 241)	<0.001
Urinary A/C (mg/g)	25.56 ± 5.14	66.31 ± 10.11	<0.001
BUN (mg/dl)	5.16 ± 1.07	6.74 ± 2.11	0.004
Cr (μmol/L)	70.01 ± 10.54	88.30 ± 22.01	<0.001
UA (μmol/L)	301.6 ± 88.5	483.7 ± 125.7	<0.001
FINS (mmol/L)	8.04 ± 1.25	8.71 ± 1. 26	0.071
FPG, SD (mmol/L)	2.05 ± 0.91	2.34 ± 0.87	0.237
FPG, CV (%)	27.34 ± 8.32	29.05 ± 6.01	0.034
MAGE (mmol/L)	5.01 ± 0.56	5.31 ± 1.34	0.073
MODD (mmol/L)	2.14 ± 0.78	2.15 ± 0.94	0.510
TAR% (>10.0 mmol/L)	24.12 ± 6.45	30.16 ± 1.29	0.003
TBR% (<3.9 mmol/L)	1.00 (0.00, 2.00)	1.00 (0.00, 3.00)	0.531
TIR (%)	75.68 ± 13.74	66.12 ± 11.87	<0.001
HbA1c (%)	7.16 ± 1.78	8.75 ± 1.91	<0.001
Use antidiabetes agents			
Oral antidiabetes drugs (%)	55.13	57.62	0.413
Insulin therapy (%)	23.52	40.13	<0.001

Abbreviations: Non-CAD: without coronary artery disease; CAD: coronary artery disease; SBP, systolic blood pressure; DBP, diastolic blood pressure; BMI, body mass index; LDL-C, low-density lipoprotein cholesterol; HDL-C, high-density lipoprotein cholesterol; ApoA: apolipoprotein A; ApoB: apolipoprotein B; ApoE: apolipoprotein E; Lpa: lipoprotein a; FPG, fasting plasma glucose; FINS, fasting insulin; SD: standard deviation; CV: coefficient of variation; urinary A/C: urinary albumin/creatinine concentration; BUN: blood urea nitrogen; Cr: serum creatinine; UA: uric acid; HbA1c, hemoglobin A1c; MAGE: mean amplitude of glycemic excursions; MODD: means of daily difference; TAR, time above range; TBR, time below range; TIR, time in range.

As shown in Table 2, TIR was 71.32 ± 18.74, 56.87 ± 15.31, and 41.57 ± 13.69 in group 1, group 2, and group 3, respectively. The severity of CAD in patients with T2DM decreases as TIR increases (Table 2, *P* < 0.01). Logistic regression analysis revealed that TIR, TAR, or CV were independently correlated with the occurrence of CAD after adjusting for confounding factors including age, sex, and BMI (Model 1) (TIR: OR = 0.924 (0.905, 0.943), *p* = 0.002; TAR: OR = 1.143 (1.102, 1.184), *p* = 0.039; CV: OR = 1.030 (1.001, 1.059), *p* = 0.025). Then, after adding diabetes duration, SBP, TG, HDL-C, and LDL-C (Model 2) or diabetes duration, SBP, TG, HDL-C, LDL-C, smoking status, use of insulin therapy, and HbA1c (Model 3) as a confounding factor, the TIR was still an independent risk factor for CAD (Table 3).

**Table 2:** Value of TIR in different GS groups

Variables	Group 1 (Gensini score ≤ 11), *N* = 112	Group 2 (Gensini score 12–36), *N* = 98	Group 3 (Gensini score > 36), *N* = 60	*P* value for trend
TIR (%)	71.32 ± 18.74	56.87 ± 15.31	41.57 ± 13.69	<0.001
HbA1c	7.37 ± 1. 56	7.91 ± 2.01	8.34 ± 1.93	0.024
CV	28.05 ± 7.11	28.21 ± 5.93	29.34 ± 7.52	0.051
SD	2.34 ± 0.73	2.33 ± 0.56	2.76 ± 1.01	0.231
MAGE	5.09 ± 1.52	5.28 ± 1.93	5.44 ± 1.07	0.054

Abbreviations: TIR, time in range; GS, Gensini score; HbA1c, hemoglobin A1c; CV: coefficient of variation; MAGE: mean amplitude of glycemic excursions.

**Table 3:** Association of TIR, TAR, and CV metrics with presence of coronary artery disease by logistic regression analyses

		Variable OR (95% CI)	*P* value
TIR	Crude	0.927 (0.912, 0.942)	<0.001
	Model 1	0.924 (0.905, 0.943)	0.002
	Model 2	0.920 (0.903, 0.937)	0.013
	Model 3	0.919 (0.896, 0.942)	0.011
TAR	Crude	1.151 (1.114, 1.188)	0.022
	Model 1	1.143 (1.102, 1.184)	0.039
	Model 2	1.056 (0.998, 1.114)	0.054
	Model 3	1.061 (0.996, 1.126)	0.055
CV	Crude	1.128 (1.017, 1.239)	0.044
	Model 1	1.030 (1.001, 1.059)	0.025
	Model 2	1.079 (1.024, 1.134)	0.046
	Model 3	1.048 (0.882, 1.380)	0.057

Abbreviations: TIR, time in range; TAR, time above range; CV: coefficient of variation; OR, odd ratio; CI, confidence interval.

Model 1 is adjusted for age, sex, and (BMI.

Model 2 includes all variables in Model 1 plus diabetes duration, SBP, TG, HDL-C, and LDL-C.

Model 3 includes all variables in Model 2 plus smoking status, use of insulin therapy, and HbA1c.

To further analyze the correlation between TIR and CAD, we conducted a binary logistic regression analysis with the severity of CAD. The results demonstrated that TIR were independently correlated with the severity of CAD after adjusting for confounding factors including age, gender, BMI, duration of diabetes, blood pressure, blood lipid, and HbA1c (Model 1) (*P* < 0.05, Table 4). This association remained significant in group 2 and group 3 after further adjustment for TAR (group 2: *p* = 0.024; group 3: *p* = 0.002) and CV (group 2: *p* = 0.037; group 3: *p* = 0.019) (Table 4).

**Table 4:** Associations between TIR and various stages of Gensini score group after controlling for confounding factor

	Non-CAD	Group 1 (Gensini score ≤ 11)		Group 2 (Gensini score 12–36)		Group 3 (Gensini score > 36)	
	OR (95% CI)	OR (95% CI)	*P* value	OR (95% CI)	*P* value	OR (95% CI)	*P* value
Model 1							
TIR	1.00 (Ref)	0.94 (0.91, 0.97)	0.003	0.96 (0.91, 1.01)	0.005	0.95 (0.92, 0.98)	<0.001
Model 2							
TIR	1.00 (Ref)	0.95 (0.89, 1.01)	0.17	0.96 (0.89, 1.03)	0.024	0.96 (0.93, 0.99)	0.002
TAR	1.00 (Ref)	1.03 (1.01, 1.05)	0.008	1.02 (0.96, 1.08)	0.145	1.13 (1.09, 1.17)	0.016
Model 3							
TIR	1.00 (Ref)	0.93 (0.83, 1.03)	0.163	0.94 (0.89, 0.99)	0.037	0.95 (0.92, 0.98)	0.019
CV	1.00 (Ref)	0.88 (0.64, 1.12)	0.41	1.28 (0.98, 1.58)	0.062	1.52 (0.97, 2.07)	0.06

Abbreviations: TAR, time above range; TIR, time in range; OR, odd ratio; CI, confidence interval; CV: coefficient of variation.

Model 1 was adjusted for age, gender, body mass index, duration of diabetes, blood pressure, blood lipid, and HbA1c.

Model 2 includes all variables in model 1 plus TAR.

Model 3 includes all variables in model 1 plus CV.

## 4 Discussion

In this study, we explored the cross-sectional relationship between TIR and CAD in patients with T2DM who underwent 72 h CGM. Our findings revealed that TIR levels were inversely correlated with the severity of CAD. Furthermore, TIR emerged as an independent determinant for the occurrence of CAD in patients with T2DM.

Diabetes is a chronic disease characterized primarily by elevated blood glucose levels [23]. The global prevalence of diabetes is expected to be as high as 10.2% in 2030, which is a significant increase from 9.3% in 2019 [24]. Notably, 90% of the diabetes cases were diagnosed as T2DM [25,26]. T2DM significantly impacts survival and quality of life, particularly in patients diagnosed at a younger age [27,28]. Although all complications of T2DM are important, cardiovascular diseases and CAD remain the leading causes of morbidity and mortality in this population [29]. Therefore, early detection and management of CAD are essential to reduce the burden of disease complications.

An increasing number of studies have shown that HbA1c alone is not sufficient to provide an optimal picture of one’s glycemic control [30,31]. With the growing use of CGM, we can now track patients’ dynamic glucose fluctuations throughout the day and obtain new glycemic management metrics. TIR has emerged as a critical metric of CGM for the assessment of short-term glucose control that has been the subject of much research interest. Previous research has established an intimate relationship between TIR and diabetic complications, such as diabetic retinopathy and microalbuminuria progression [18,32]. Recently, Lu et al. demonstrated that TIR was inversely correlated with intima-media thickness in the carotid arteries in T2DM, highlighting a potentially plausible link between TIR and diabetic macrovascular complications [33]. Our previous work that showed TIR was an independent protective factor for diabetic peripheral artery disease also lends support to this relationship. Therefore, we believed that TIR may potentially serve as a research endpoint in T2DM-related clinical trials [34]. To the best of our knowledge, the correlation between TIR and CAD has not been investigated previously. In the current study, we found that patients with T2DM and CAD had significantly lower TIR compared to those without CAD (66.12 ± 11.87 vs 75.68 ± 13.74). Moreover, CAD severity as indicated by the Gensini score tends to decrease with total internal reflection. T2DM patients with different CAD severities were found to have dramatically disparate TIR values and the multivariable-adjusted logistic regression analysis supported that TIR was an independent determinant for CAD occurrence in patients with T2DM.

CAD is influenced by multiple factors. Dyslipidemia is a well-recognized risk factor for CAD across various populations [35]. Tseng identified that age, hypertension, HDL-C, and percent body fat are independently associated with CAD [36]. Similarly, Bittencourt et al. demonstrated that glucose and HbA1c levels correlate with the extent and severity of coronary artery lesions [37]. Therefore, when assessing the relationship between TIR and CAD, we adjusted for potential confounding factors. After these adjustments, the independent association between TIR and CAD remained significant, indicating that the relationship between TIR and CAD is not influenced by other risk factors such as age, gender, BMI, duration of diabetes, blood pressure, blood lipid levels, and HbA1c.

This study suffers from several limitations. First, the study was a cross-sectional design and could not establish a causal relationship between TIR and CAD. Second, despite adjusting the models for numerous factors, residual confounding may still exist. Additionally, the study population consisted solely of hospitalized T2DM patients, excluding non-hospitalized individuals, which may limit the generalizability of the findings. Therefore, the results should be interpreted with caution. Third, this study did not compare the predictive value of TIR and HbA1c for the severity of CAD. Fourth, we did not explore whether TIR can be practically implemented in routine clinical practice or whether specific TIR thresholds can guide treatment decisions for CAD in patients with T2DM. To address these limitations, future research with greater precision is necessary.

In conclusion, our findings provide evidence that TIR is associated with the severity of CAD in patients with T2DM and serves as an independent prognostic factor for CAD. We suggest that TIR should be more widely accepted as a research endpoint or clinical measure. However, further studies, ideally prospective in nature, are needed to confirm our findings and to better characterize the role of TIR in relation to CAD severity in T2DM patients.
